# A manifesto for the future of ICU trials

**DOI:** 10.1186/s13054-020-03393-5

**Published:** 2020-12-09

**Authors:** Ewan C. Goligher, Fernando Zampieri, Carolyn S. Calfee, Christopher W. Seymour

**Affiliations:** 1 Interdepartmental Division of Critical Care Medicine, University of Toronto, Toronto, Canada; 2 Division of Respirology, Department of Medicine, University Health Network, Toronto, Canada; 3 Toronto General Hospital Research Institute, 585 University Ave., 11-PMB Room 192, Toronto, ON M5G 2N2 Canada; 4 Research Institute, Hcor-Hospital Do Coração, São Paulo, Brazil; 5 Division of Pulmonary, Critical Care, Allergy and Sleep Medicine, University of California San Francisco, San Francisco, CA USA; 6 The Clinical Research, Investigation, and Systems Modeling of Acute Illness (CRISMA) Center, Department of Critical Care Medicine, University of Pittsburgh School of Medicine, Pittsburgh, PA USA

The intensive care unit (ICU) is both a challenging and opportune environment for the conduct of clinical trials. On the one hand, competing determinants of patient outcome (including multi-morbidity and pre-ICU illness trajectory) and the heterogeneity of critical illness syndromes attenuate the population-average treatment effect [1, 2]. On the other hand, the ICU is a controlled environment that facilitates monitoring of protocol adherence and outcome ascertainment. ICU trials may be improperly powered because of overly optimistic assumptions about the baseline event rate in the control group and about the predicted effect of treatment on that event rate [3, 4]. The treatment effect required to demonstrate statistically significant benefit often substantially exceeds what might be considered the minimum clinically relevant benefit, and consequently, trials sometimes are interpreted to show “no evidence of benefit” even when clinically relevant benefits are observed.

The COVID-19 pandemic has shown that we need to (and can) find a way to deliver more effectively on trials in the ICU. The benefit of dexamethasone was demonstrated within just a few short months of the outbreak of the global pandemic [5]. Conversely, many tens of thousands of patients were treated with unproven and potentially harmful therapies outside of trials, and the benefit of certain interventions remains uncertain due to the challenges of completing trials of these rapidly adopted therapies.

We therefore propose a manifesto for the future of ICU trials (Table 1).

1. *Think Bayesian* Bayesian analysis is an alternate statistical paradigm that answers the question “what is the probability of treatment effect” in contrast to the traditional frequentist approach, which answers the question “what is the probability of these data, assuming no treatment effect?” Under the Bayesian framework, trial information is not biased by “looking at” the data, and the results can be continuously re-estimated and updated as additional information (i.e., patient outcomes) is added to the dataset [6]. To put it simply (and perhaps somewhat simplistically), conventional frequentist statistics views the entire trial as a single “coin flip”; technically, there is no information to draw conclusions until the trial is completed. By contrast, Bayesian statistics regards each individual patient’s outcome as a “coin flip”; the estimated probability of benefit or harm can be continuously updated as information accumulates. We contend that the Bayesian approach is ideal because it (a) directly answers the questions of interest (probabilities of clinically relevant benefit, harm, or futility), thereby reducing the risk of a false “positive” or false “negative” conclusion; and (b) the continuously updated posterior permits maximally efficient trial adaptations in sample size and treatment allocation [7].
2. *Adapt when needed* Most trials in COVID-19 adopted an adaptive trial design given deep uncertainty about actual event rates and treatment effects. Adaptive designs respond flexibly to observed event rates and treatment effect, avoiding the risk of underestimating sample size requirement because of overly optimistic predictions about event rates and treatment effect [8]. Adapting treatment allocation probabilities within the randomization algorithm (response-adaptive randomization) can also increase trial efficiency in trials with three or more arms by dropping poorly performing interventions and identifying treatment-responsive subgroups earlier (rather than waiting until the end of the trial to draw a conclusion).
3. *Build* *a platform* Platform trials leverage the infrastructure for recruitment, treatment allocation, outcome ascertainment, and analysis to evaluate multiple interventions for a single disease state or multiple disease states [8]. Just as “multiple games” can be played in a single “stadium,” multiple trials—including Phase II, III, or IV trials—can be run sequentially or concurrently on a single platform. RECOVERY and REMAP-CAP provide important examples of phase III platform trials in COVID-19 [9, 10]. I-SPY2 provides a useful example of a Phase II platform intended to test many potential interventions for breast cancer, prioritizing the most promising agents for Phase III trials [11]. I-SPY2 investigators have now teamed with intensivists to launch an adaptive platform trial (I-SPY COVID) for severe COVID-19, with a similar Phase II focus.
4. *Understand the noise* In critical illness syndromes, the specific biological and physiological mechanisms driving outcomes may vary considerably (“noise”) between patients. Hence, the benefit of therapeutics (“signal”) targeting those mechanisms will also vary. To increase the probability of demonstrating the benefit of therapy in treatment-responsive subgroups (where such benefit actually exists)—to find the signal in the noise—heterogeneity in treatment response needs to be characterized as much as possible before and during Phase III trials [12, 13]. Adaptive trials can be designed to take account of relevant biological/physiological heterogeneity and to facilitate the discovery of such heterogeneity during the trial [11].
5. *Be inclusive* To ensure that trial results are truly generalizable, we need to ensure that appropriately diverse and representative patient populations are enrolled in clinical trials. Perhaps the best way to achieve this is to cast the widest possible net so that nearly every critically ill patient (including those outside traditional academic centers) has the opportunity to participate in a trial.
6. *Embed discovery within care* Trials are costly, data collection is labor-intensive, and finding patients can be difficult. Embedding trials—both electronically and culturally—offers a solution. Embedding trials within existing data repositories (e.g., clinical registries, electronic health records) to “find” patients, randomly assign treatments, collect data, and ascertain outcomes can increase efficiency and reduce costs. Trials can be embedded within the culture of clinical practice to achieve continuous quality improvement through discovery and innovation, an approach referred to as “learning while doing” [14]. Utilizing the uncertainty of clinical decision-making as an opportunity for randomization could dramatically accelerate our capacity to improve care and outcomes for patients [15]. The culture of ICU healthcare delivery needs to increasingly see clinical trials as part of its core mission to deliver the very best possible care for patients. Trials are not ancillary to high-quality patient-centered care—they are integral to the mission.

**Table 1:** Challenges and opportunities for clinical trials in critical care

Proposal	Current state	Barriers to implementation	Potential solutions
Think Bayesian	Nearly all clinical trials are designed based on frequentist statistics, which yields less information about the probability of benefit and harm than Bayesian statistics	Unfamiliarity with the Bayesian framework and adaptive trial design Skepticism about the perceived subjectivity of Bayesian prior distributions	Education for clinicians, investigators, and funders Widespread appreciation for the close analogy between Bayesian statistics and routine clinical reasoning
Adapt when needed	Standard clinical trials employ rigid designs with fixed sample sizes based on educated estimates of event rates and treatment effect	Skepticism about the statistical validity of frequent interim analyses to guide adaptations in design Methodological expertise in adaptive trial design and Bayesian trial design is not yet widespread	Dissemination of consensus on best practices for the design and conduct of adaptive trials Successful completion and publication of arms and domains from ongoing adaptive trials
Build a platform	Traditional clinical trials typically build trial infrastructure (funding, regulatory approval, logistics, recruitment, investigator network, analysis, data monitoring committee) to address one research question	Funding is complex: Both the platform infrastructure and the individual interventional research questions require funding support Authorship criteria can be challenging to establish	Proactive partnerships with funding agencies to support platform infrastructure Academic community should intentionally place greater value on group authorship
Understand the noise	Some trials are criticized for being too pragmatic and providing fixed interventions regardless of mechanistically relevant physiological or biological characteristics of individual patients	Demonstrating heterogeneity of treatment effect increases sample size requirements Clinical importance of different mechanistic pathways may not be clear prior to the conduct of the trial	Development and validation of short-term surrogate endpoints reflecting mechanistically relevant treatment response for phase II trials (similar to phase II oncology trials) before evaluation in phase III trials Use of response-adaptive randomization and other adaptive trial techniques to enhance recognition of differential treatment response among relevant patient subgroups
Be inclusive	Most patients in critical care units are not enrolled in a clinical trial Many trials do not include patients from resource-constrained settings	Restrictive inclusion and exclusion criteria Challenges of obtaining informed consent in timely fashion for time-dependent interventions	Create potential incentives to including patients in clinical trials Consider enrolment in trials as a quality performance marker for healthcare systems Enhance patient engagement in trial design
Embed discovery within care	Trials employ dedicated electronic case report forms and data coordinating centers Data entry is often duplicated across multiple interfaces, increasing both workload and errors	Widely accepted dissociation between clinical care and clinical research. Widespread variability in electronic health record systems Resource-constrained settings may not have access to electronic health record systems Algorithms for detecting and reducing missing data are not routinely implemented outside of trials	Partner with healthcare administrators to design trials that contribute to quality and innovation within healthcare systems Integrate clinical record-keeping with minimum datasets for clinical research Develop low-cost electronic health record systems for widespread use

## Data Availability

Not applicable.
